# Institutionalizing human-computer interaction for global health

**DOI:** 10.1080/16549716.2017.1344003

**Published:** 2017-08-25

**Authors:** Jan Gulliksen

**Affiliations:** ^a^ KTH Royal Institute of Technology, School of Computer Science and Communication, Department of Media Technology and Interaction Design, Stockholm, Sweden

**Keywords:** mHealth for Improved Access and Equity in Health Care, Human-computer interaction, digitalization, action research, health care, global health

## Abstract

Digitalization is the societal change process in which new ICT-based solutions bring forward completely new ways of doing things, new businesses and new movements in the society. Digitalization also provides completely new ways of addressing issues related to global health. This paper provides an overview of the field of human-computer interaction (HCI) and in what way the field has contributed to international development in different regions of the world. Additionally, it outlines the United Nations’ new sustainability goals from December 2015 and what these could contribute to the development of global health and its relationship to digitalization. Finally, it argues why and how HCI could be adopted and adapted to fit the contextual needs, the need for localization and for the development of new digital innovations. The research methodology is mostly qualitative following an action research paradigm in which the actual change process that the digitalization is evoking is equally important as the scientific conclusions that can be drawn. In conclusion, the paper argues that digitalization is fundamentally changing the society through the development and use of digital technologies and may have a profound effect on the digital development of every country in the world. But it needs to be developed based on local practices, it needs international support and to not be limited by any technological constraints. Particularly digitalization to support global health requires a profound understanding of the users and their context, arguing for user-centred systems design methodologies as particularly suitable.

## Background

In today’s rapid development of the society, digitalization is the new concept used to capture the transforming powers that technology brings, a power by which new competitors in the Western world may shake the ground for well-established institutions. In developing countries, the rapid development of new technology may mean that completely new applications, use patterns and societal changes may emerge. Particularly it is societal digitalization that is mostly of interest, and we use the following definition: ‘societal digitalization – the social and human-disruptive process that gradually becomes increasingly difficult to distinguish at all from any part of life. This means that individuals and organizations can communicate and exchange information with other people, organizations and their environment in new ways. Digitalization and the use of ICT-based solutions can help to increase the accessibility and efficiency of both the business and public administration’ [1].

**Figure 1.**
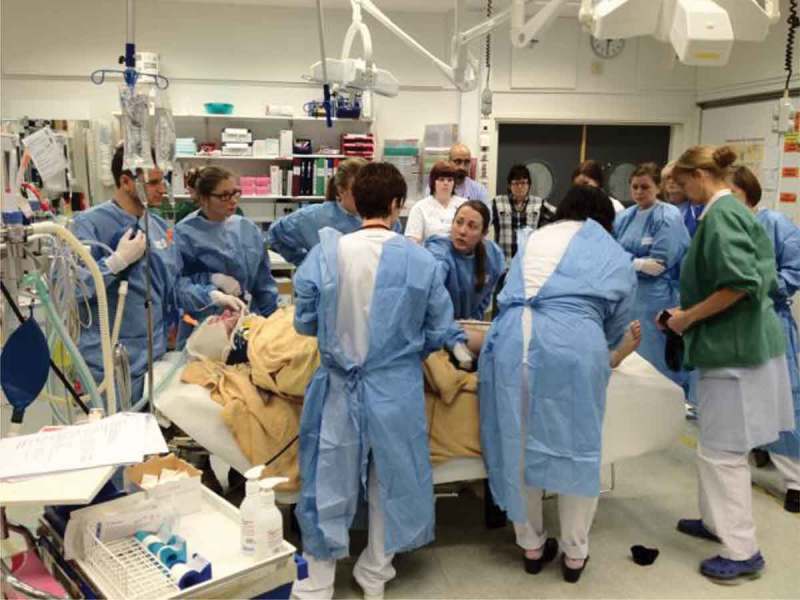
The use of mobile digital tools in a trauma training facility.

## Human-computer interaction

Human-computer interaction (HCI) is a discipline concerned with the design, evaluation and implementation of interactive computing systems for human use and with the study of major phenomena surrounding them [2]. Given the field’s close collaboration and interaction with users it has been a central field within ICT for development, which is a field of its own (ICT4D: ICT for development in developing countries) [3]. Hence it is natural to see that most of the research within the field of ICT for development has happened within the field of HCI. Additionally, most of the program code that is written deals with the user interface, so it is a substantial part of the computing field. When working within human-computer interaction the quality of the interaction is important to consider and therefore the concept of usability becomes important. Usability can be defined as the extent to which a product can be used by specified users to achieve specified goals with effectiveness, efficiency and satisfaction in a specified context of use [4]. To be able to develop usable systems consciously one needs to adopt a user-centred design process. User-centred systems design can be defined as a process focusing on usability throughout the system life cycle and it is based on 12 key principles [5].

In the past, we have been working with the goal of introducing HCI and interaction design in new places in the world, such as India [6] and China [7]. From our previous projects we have seen that a successful institutionalization of usability and HCI in developing countries requires three elements: firstly an appropriation of HCI concepts and methods to suit the local country/culture, secondly the forming of a national organization around the reshaped discipline that can actively promote HCI in industry and academia and establish links with local national organizations, and thirdly the roll-out of effective usability practice in industry [8]. Adapting methods and tools to local needs and requirements requires both a deep understanding of the local culture and development routines as well as a genuinely collaborative development approach [9].

## United Nations sustainability goals

In February 2015, many of the world’s countries agreed to work towards meeting the new sustainability goals. They were based on the issues argued by Griggs et al. [10]. These goals set new and much higher goals for societal development than the previous millennium development goals (MDG) [11]. The sustainable development goals include issues of eradicating poverty, eliminating diseases, promoting global education for everybody, social inclusion and access to digital technologies and the internet. To be able to meet these goals of societal impact we need to work tirelessly with the goals of understanding the challenges of digitalization in developing nations and with the aim of adapting technical design and development methodologies to fit the needs and goals of development under these circumstances. Or as Hugo [12] puts it: ‘In multicultural environments it is even more important [to] consider how our understanding of the complex dialectic between culture, economy and technological innovation influences our ability to empower our people’ (p. 4).

One of the major sustainability challenges to work with is the issues concerning global health and the impacts new digital technologies may potentially have. Digitalization is changing how health care can be conducted all over the world. The Internet has provided access to enormous amounts of information about health care, diseases and medical support. It is now quite common that a patient before a visit to the doctor has studied a lot of information about their condition through various Web-based tools. The role of the medical doctor, in times when the patient may be more up to date on recent information about their condition, will change from being the highly valued and undisputed expert to being a person helping the patient interpret and value the obtained information. Mobile technology and easy-to-use communication software facilitate remote communication and judgements using these media. A high-quality camera may provide opportunities to take pictures of injuries or skin conditions that can be communicated remotely to allow doctors to judge the injuries and support the patient throughout the treatment. Through the ‘quantified self’ movement patients share enormous amounts of health data and can thus facilitate data science, for example for overviewing and following the spread of a disease. GPS and geographical localizations can aid the doctor in locating the spread of a disease.

This is, however, not without problems. Digital technologies have many positive aspects but also one needs to be aware of and plan to overcome the challenges related to security, privacy and integrity. We need to closely monitor and adapt liability issues when conducting care online through digital tools. And we also need to investigate usability and accessibility issues to manage a healthy development. Particularly when it comes to medical and health-related applications it is of the utmost importance that the usability is fully functioning as it otherwise could jeopardize people’s lives.

## The Stockholm Declaration for Global Health

The Stockholm Declaration for Global Health [13] declared in 2015 the goal of promoting social justice globally and of safeguarding the wellbeing of current and future generations. The Stockholm Declaration for Global Health urges governments, the global health community, schools and universities, development agencies, donors, policy-makers, research funding agencies, the business sector and civil society to act urgently on existing evidence in the following areas:  

- **Linking on-going agendas with new agendas.**Ensure that the post-2015 development agenda builds on current MDGs, is universal and incorporates emerging challenges. These include socioeconomic and gender inequalities, non-communicable diseases (such as heart disease, stroke, diabetes, cancer and chronic respiratory disease) and climate change (including threats to food and water security).
- **Creating stronger leadership and accountability so that health is at the centre of development**. Ensure that health is a high-profile unifying theme in the post-2015 development agenda, positioned to act as a catalyst for human rights and global solidarity, and that appropriate accountability mechanisms and professional leadership for global and national commitments are established.
- **Building capacity and investing in health**. Invest in leadership for global health through education from primary school to university, and enable public empwerment by bringing together networks for intersectoral multidisciplinary research and action on global health.
- **Exploiting opportunities and synergies**.Identify and exploit opportunities for applying effective democratic principles to ongoing health agendas (including maternal, child and mental health), violence, climate change and other emerging challenges, thus bringing sustainable social, ecological and economic short-term and long-term returns for both public and private sectors. Pursue synergies such as health and climate co-benefits that bring multiple gains.

But, as mentioned before, digitalization is the biggest societal transformation process that every sector of society currently undergoes. This is also very much the case for the health-care sector. Digitalization makes it possible to do things in new ways, and it makes it possible to do new things.

## Action research to promote change

To understand a complex societal process of change, such as understanding the effects of a software development project in context, involving many people, both online and offline, requires a qualitative research approach. There are few measurable qualities that could be perceived as important to judge the development and progress. Additionally, we aim to conduct the research with the explicit objective of improving the society and thus we are not independent observers of a process, but, as researchers, active participants in a process of societal change. The appropriate overarching research methodology is therefore ‘action research’, in which the actual changes which the research is able to contribute to in society are of equal importance to the scientific observations, and the mere fact that we conduct the research with a change agenda is an asset in the process [14].

In action research, we use qualitative data-gathering methods, such as interviews, observations, workshops etc., and holistically analyse all information, acknowledging the quality criteria for social research proposed by Klein and Myers [15].

Since the research is about contributing to change we apply design methods as a tool for the research [16].

## Examples of digitalization efforts within health care

There are many areas in which digital technologies may have the potential to improve health care. From our and others’ research as well as innovations in society we have seen many recent development efforts and have been able to draw conclusions based on the effects that these development efforts have had. In the following we mention a few of the results that we think have a bearing on the digitalization of health care for developing countries:

- **Digital technologies for preventive health care –** Digital technologies may prove to be very useful for spreading information and knowledge for preventive health care: for example, using technologies for health campaigns to promote preventive health care [17]. Digital technologies can help people who suspect they have some form of disease to get more information about their symptoms and condition through online dictionaries and knowledge databases. They can also get information from social networks dedicated to patients who have gone through similar situations and how they have dealt with such types of situations. One such popular service is Patientslikeme®.
- **Digital technologies to make administration more effective –** Digital tools may help planning health consultations and schedule visits better for increased efficiency, and can facilitate transfer between different ward units and ensure synchronization between different care-givers. They may be able to create an overview of the patient’s healthcare plan. Additionally, they may provide opportunities for health-care workers to establish a more effective work situation. Studies of the implementation of a surgical planning in Sweden confirms the above-presented improvements. The ICT system for planning surgery did increase the overview, and helped the planners with more and more relevant information about the surgery.
- **Digitalizing the medical record –** Digital medical records create more solid patient information that can be accessible from many different places. Providing patients with Web-based access to their own medical records has proved to be useful and appreciated by patients. A study with cancer patients in Sweden showed that some patients preferred to receive their diagnosis by reading about it in their medical record instead of having to wait for the information and to be able to process it emotionally before seeing their doctor in order to be able to ask questions [18]. Considering the often short time available for consultation with a doctor, accessing the medical record enables patients also to prepare themselves for an upcoming visit and can serve as a memory aid for what has been discussed during the visit [18].

- **Digital skills for information and communication –** Digital technologies may help people to build and deepen their skills and collect information on their health condition. At the same time, engaging with these technologies requires a skill set which has been termed eHealth literacy [19]. The lack of eHealth literacy skills bears the risk of extending the digital divide into the healthcare domain [20], which has to be addressed accordingly. A survey of patients reading their medical records online in Sweden confirms that the users of the system has a very high level of education, and that many users work in health care [21]. This indicates that the users of the system have good eHealth literacy.
- **Digital technologies for remote asynchronous medical judgements –** Digital technologies may help individuals to do self-diagnosis and provide remote asynchronous medical judgements. In rural areas, it may be possible to take pictures and communicate remotely, synchronously or asynchronously, with doctors, to get initial recommendations or advice, to help in traumatic situations when no doctors have a chance of getting in immediate touch. One can conclude that while eHealth interventions are rare in constrained areas, mHealth is widely used for care and management [22].

- **Sensor technologies and remote monitoring –** Sensor technologies allow remote and continuous monitoring of patients who agree to sharing their data. This has been shown to be particularly interesting in relation to the care of older patients. Sensor technologies can show movement and location and thus help tracing patients or keeping track of their movements during the day. The EU Ambient Assisted Living project made great use of these technologies. Research in Austria has, however, shown that the development of sensor-based systems for older people requires a thorough understanding of this diverse user group. It is challenging to successfully implement sensor-based technology despite its great potential [23].
- **Digital resources in trauma and disasters –** Digital tools may provide help when access to medical professionals is scarce, particularly when accidents or bigger disasters happen. Digital technologies may be very useful during rehabilitation and training to regain strength after a trauma. Handling specific traumas in intensive care is a complex task where digital technologies can, if used in the right way, mean the difference between life and death. In a forthcoming PhD thesis (Alexander Yngling, KTH), the design of mobile technologies to aid doctors and nurses of different specializations in trauma has been tested (Figure 1).
- **Digital technologies for medication –** Digital technologies may help patients remind themselves about medications and provide pharmaceutical information [24]. Many people need specific assistance to be able to use the right medication. Taking a too small or too big dose of a prescription is far too common and could easily be prevented if patients have up-to-date digital tools that can help them in their daily medication.

These examples serve the purpose of showing experiences obtained by conducting action research projects in medical settings, and/or digital innovations for health and care. The experiences obtained can of course not be generalized to any setting, and particularly not to an African setting as the technology availability, the maturity of digital skills, the infrastructure and the culture of use is very different. However, they all show that innovations relating to mobile health or the use of digital technologies to improve health and care all teach us important things about the process of designing and introducing these technologies. They show us the importance of considering the end-users of the technologies and their context of use, all in line with the general approaches in the field of human-computer interaction. They show that the concept of digitalization brings much more than just introducing new technologies in an existing setting: it is more about the change in business and practices through the use of the technology instead of just the technology itself. Also, it shows that action-research-oriented methodologies serve the purpose of conducting research in a setting where the main goal is the societal change that the technology invokes.

## Discussion

Institutionalizing human-computer interaction for global health care requires changing the views on systems development in industry, changing the policy-making and strategies to reflect this and changing the way in which we teach and educate about the subject.

- **Digital skills for improved health and care –** The need for increased digital skills for everybody should be a top priority for every country. Facilitating learning through the active use of digital technologies at all ages is important for the digitalization of society. Education plays an increasingly important role in improving the health care.
- **Digital health and care jobs –** The need for highly educated ICT competencies is acute and increasing. The digital sector is the fastest-growing employment sector with negative unemployment worldwide. It is a source of entrepreneurship and innovation and requires a competence shift in the workforce of today. It is particularly growing in the non-ICT sector, such as the medical sector.
- **Access to hardware and infrastructure supporting health and care –** By making the price of digital technologies sufficiently low and by making the Internet infrastructure accessible, stable and reliable, the opportunities to make use of this infrastructure for democratization, skills development and digital transformation for health and care increase.

The development and uptake of different technologies may look very different in different parts of the world, because of availability, costs and technical readiness. The Western world has seen a development from stationary computers, through laptop computers to tablets and mobile computers, now moving towards the Internet of things, sensors and ubiquitous technologies. It has gone from professional work-related use to more private and leisure use. Many countries from the developing world skip one or more steps in this process, providing access to mobile technologies with no or little use of stationary or laptop computers. Mobile technologies are cheaper, safer and easier to use in a more personalized manner.

The ways of using the digital technologies vary a lot in different parts of the world due to culture, but also to availability. In the Western world, a mobile phone may be a very private gadget that one rarely gives to someone else, but in rural Africa a mobile phone may be shared by many in a greater social context.

Through the increasing digitalization of higher education and the increasing international availability through MOOCs and other digitally available technologies for teaching and learning, the opportunities for building skills and jobs in the developing countries as well increase tremendously. Such a technological development will, if properly used, play an important role in democratization and equitable opportunities for development and in contributing to several of the sustainability development goals.

It is necessary to develop and adapt the methodologies and processes for design and development of digital transformation to the specific regional conditions in each respective area. By opening up development, crowdsourcing development to allow for many to contribute, by being more agile and allowing for changing requirements development could be made much more relevant for other parts of the world. Changing behaviour and attitudes to allow a more user-centred approach, however, has so far turned out to function well in different parts of the world, even if the ways in which it has been adopted change in different regions [8].

There is also a risk for ‘imperialistic colonialization’ of software development in which ‘digital superpowers’ impose their development cultures without acknowledging the local aspects in the interest of digitalization. Supporting international development and digitalization requires a fair bit of humility and respect for local traditions and cultures to manage a healthy introduction of new development processes, tools, practices and methods. Moreover, research has shown that the introduction of new technology in health care is sometimes met with resistance and that inertia is strong when it comes to the establishment of technology [21]. When online medical records were introduced in Sweden physicians strongly disapproved of the system as they were worried about their work environment and about patients reading medical records online and becoming worried about what they read [25].

In conclusion, digitalization is fundamentally changing society through the development and use of digital technologies. It may have a profound effect on the digital development of every country in the world. But it needs to be developed based on local practices, it needs international support and not to be limited by any technological constraints. There is a need for a responsible design process that involves an understanding of local cultures and traditions for healthy international systems development and digitalization. Particularly digitalization to support global health requires a profound understanding of the users and their context and the entire health-care system and thus requires a user-centred systems design approach.

## Paper context

Based on our extensive experiences of digitalization and studies of human computer interaction both in health and care and in other domains, and using our experiences from bringing these subjects forward to developing countries I have reasoned about the experiences drawn and what implications these experiences potentially can have when bringing it to developing countries. We argue for an action-oriented research paradigm using user-centred design methodologies to institutionalize user-centred systems design for global health.
